# Clinical Efficacy and Safety of Shensong Yangxin Capsule-Amiodarone Combination on Heart Failure Complicated by Ventricular Arrhythmia: A Meta-Analysis of Randomized Controlled Trials

**DOI:** 10.3389/fphar.2021.613922

**Published:** 2021-02-22

**Authors:** Xinfu Cao, Mingxue Zhou, Hongxu Liu, Xiufen Chen, Xiang Li, Sihan Jia

**Affiliations:** ^1^ Department of Cardiology, Beijing Hospital of Traditional Chinese Medicine, Capital Medical University, Beijing, China; ^2^ Beijing Hospital of Traditional Chinese Medicine, Capital Medical University, Beijing Institute of Traditional Chinese Medicine, Beijing, China; ^3^ School of Chinese Materia Medica, Beijing University of Chinese Medicine, Beijing, China

**Keywords:** Shensong Yangxin capsule, traditional Chinese medicine, amiodarone, heart failure, Ventricular arrhythmia, meta-analysis

## Abstract

**Background:** Shensong Yangxin capsule (SSYX) is a well-known traditional Chinese patent medicine for treating arrhythmia. Recently, a flurry of randomized controlled trials (RCTs) of SSYX combined with amiodarone (SSYX-amiodarone) was reported in the treatment of heart failure (HF) complicated by ventricular arrhythmia (VA) in China. However, these RCTs have not been systematically evaluated. Therefore, this study aimed to evaluate the efficacy and safety of SSYX-amiodarone in the treatment of heart failure complicated by ventricular arrhythmia (HF-VA).

**Methods:** Seven electronic literature databases (the Cochrane Library, PubMed, EMBASE, China Biomedical database web, China National Knowledge Infrastructure Databases, Chinese Scientific Journal database and Wanfang database) were searched from their inceptions to June 1, 2020 to identify RCTs of SSYX-amiodarone in the treatment of HF-VA. The primary outcomes included the total effective rate and adverse events (ADRs). The secondary outcomes included the frequency of ventricular premature complexes, left ventricular ejection fraction, N terminal pro Btype natriuretic peptide (NT-proBNP), and QT dispersion (QTd). The quality of the included RCTs was assessed using the Cochrane risk-of-bias tool. All data was analyzed using RevMan 5.3 software. The registration number of this protocol is PROSPERO CRD42020196689.

**Results:** There are Eighteen trials involving 1,697 patients were included in this study. Meta-analysis showed that SSYX-amiodarone group was superior to the amiodarone group in improving the total effective rate [RR = 1.21; 95%CI (1.16, 1.27); *p* < 0.01], meanwhile reducing the ADRs [RR = 0.65; 95%CI (0.45, 0.95); *p* = 0.03], VPCs [MD = 170.96; 95%CI (159.88, 182.04); *p* < 0.01] and QTd [MD = 8.39; 95%CI (6.91, 9.87); *p* < 0.01]. No significant difference of enhancing LVEF [MD = 4.32; 95%CI (−0.56, 9.20); *p = 0.08] and reducing NT-proBNP [SMD = 0.17; 95%CI (−0.81, 1.14); *p* = 0.73] was observed between SSYX-amiodarone and amiodarone group*.

**Conclusions:** Despite the apparent positive findings reported, the evidence provided by this meta-analysis was still insufficient to support the routine use of SSYX-amiodarone for HF-VA due to the poor methodological quality of included studies. The overall effect should to be verified in further through more well-design clinical studies with reasonable sample and good methodological quality.

## Introduction

Heart failure (HF) is the end stage of various heart diseases, affecting the total 1–2% population in United States (Mozaffarian et al., 2016). Currently, the HF incidence rate is 0.9% in China (Chen et al., 2017), and 50% of HF patients have sudden cardiac death, which is closely related to ventricular arrhythmia (VA) (Gust et al., 2005). HF and VA often occur at the same time, and almost all patients with HF can record VA in the dynamic electrocardiogram monitoring (Santangeli and Marchlinski, 2015). HF and VA promote each other, HF will increase the incidence of VA, and VA can also enhance the severity of HF and increase the risk of HF-induced death (Leslie et al., 2006; Goldberger et al., 2011).

Amiodarone, which exerted anti-arrhythmic effect through prolonging action potential duration of cardiomyocytes, played an extremely important role in treating HF complicated by VA (HF-VA) (Li et al., 2019). While amiodarone had many adverse reactions (ADRs), including bradycardia, atrioventricular block, QT interval prolongation, pulmonary toxicity, hepatotoxicity, thyroid dysfunction, skin changes, and so on (Jaworski et al., 2014; Danzi and Klein, 2015), which have limited the wide usage of amiodarone in clinic. Nowadays, although Non-drug therapies such as implantable cardioverter defibrillator implantation, radiofrequency catheter ablation, and cardiac resynchronization therapy have been widely used in the treatment of HF-VA, a considerable number of HF-VA patients were still unable to get effective treatments (Sardu et al., 2017). Hence, it was much-needed to explore some other effective interventions for treating HF-VA. Some studies (Ju et al., 2019; Peng et al., 2020) indicated that, as a kind of ethnic medicine, traditional Chinese medicine has potential advantages in the treatment of HF and VA.

Shensong Yangxin capsule (SSYX) is a kind of Chinese patent medicine, widely using in the treatment of arrhythmia in China (Li, 2020). It composed of Ginseng Radix et Rhizoma, Radix Ophiopogonis, Corni Fructus, Taxilli Herba, Eupolyphaga, Paeoniae Radix Rubra, Coptidis Rhizoma, Schisandrae Sphenantherae, Os Draconis. SSYX has the functions of tonifying Qi and Yin, promoting blood circulation to remove meridian obstruction, clearing away heart fire, and calming the mind. The overall ingredient of SSYX was listed in Table 1.

**TABLE 1:** Overview ingredient of Shensong Yangxin Capsule.

Chinese name	Pharmaceutical name	Species	Family
RenShen	Ginseng Radix et Rhizoma	Panax ginseng C.A.Mey	Araliaceae
MaiDong	Radix Ophiopogonis	Ophiopogon japonicus (Thunb.) ker gawl	Liliaceae
ShanZhuYu	Corni fructus	Cornus oj-jZcinalis sieb. etZucc	Cornaceae
SangJiSheng	Taxilli herba	Taxillus chinensis (DC.) danser	Loranthaceae
TuBieChong	Eupolyphaga	Eupolyphaga sinensis walke	Eupolyphaga
ChiShao	Paeoniae Radix Rubra	Paeonia veitchii lynch	Ranunculaceae
HuangLian	Coptidis Rhizoma	Coptis chinensis franch., coptis deltoidea	Ranunculaceae
NanWuWeiZi	Schisandrae sphenantherae	Schisandra sphenanthera Rehder and E.H.Wils	Magnoliaceae
LongGu	Os draconis	Fossils of bones of elephant, rhinoceros or horse	-

In recent 10 years, a flurry of randomized controlled trials (RCTs) of SSYX combined with amiodarone (SSYX-amiodarone) in the treatment of HF-VA was reported in China. These RCTs showed that SSYX-amiodarone could not only improve the efficacy in the treatment of HF-VA compared with amiodarone alone, but also reduce the incidence of ADRs (Zhao, 2018; He, 2019). However, there was insufficient evidence to support the conclusion. Therefore, we conducted a meta-analysis to evaluate the efficacy and safety of SSYX-amiodarone in the treatment of HF-VA.

## Material and Methods

This study was conducted following the registered protocol with PROSPERO (Protocol number: CRD42020196689) (Cao et al., 2020b). This meta-analysis was performed in accordance with The Preferred Reporting Items for Systematic Reviews and Meta-Analyses (PRISMA) guideline (Moher et al., 2015). A completed PRISMA checklist is included as an additional file (Supplementary File 1).

### Literature Search

Literature searching was performed in several databases of Cochrane Library, PubMed, EMBASE, China Biomedical database web, China National Knowledge Infrastructure Databases, Chinese Scientific Journal database and Wanfang database from their inceptions to June 1, 2020. A list of Chinese and English journals that might publish potentially eligible studies were also searched manually. The details of search terms and strategies are described in the Supplementary Files 2.

### Study Selection

The criteria about literature inclusion are as follows: 1) Study design: RCTs. 2) Participants: All the enrolled participants were required to meet the current or past definitions of HF and VA. Trials without a description of the detailed diagnostic criteria but which reported patients with definite HF-VA were also included. 3) Interventions: The control group was treated with amiodarone alone. While, the experimental group was treated with SSYX and amiodarone. The basic therapies in two groups were similar, including angiote nsin II receptor antagonists, angiotensin-converting enzyme inhibitors, beta-blockers, diuretics, aldosterone receptor antagonists, nitrates, et al. SSYX was the only intervention difference between the control group and the experimental group in all studies. 4) Outcomes: The primary outcomes included the total effective rate and ADRs. The secondary outcomes included the frequency of ventricular premature complexes (VPCs), left ventricular ejection fraction (LVEF), N terminal pro Btype natriuretic peptide (NT-proBNP), and QT dispersion (QTd). The included studies reported at least one of the above outcomes.

The criteria of literature exclusion were as follows: 1) For duplicate publications, only those with the earliest publication time were selected. 2) Interventions in the control group included other traditional Chinese medicine therapies, such as acupuncture or Chinese herbs. 3) The criteria of efficiency evaluation did not meet the following definitions: Markedly effective: Based on 24 h electrocardiogram, VPCs disappeared or decreased by 70% or more; Ventricular tachycardias (VTs) disappeared or decreased by 80% or more; The improvement of New York Heart Association functional class (NYHA) was more than two grades or reached grade I, and there was an obvious improvement in the clinical symptom. Effective: VPCs decreased by 50–70%; VTs decreased by 50–80%; The improvement of NYHA was more than one grade or reached grade I, and the clinical symptom partially improved partly. Ineffective: It did not reach the above standards of efficiency, and even exacerbation. The total effective rate = markedly effective rate + effective rate.

### Data Extraction and Risk of Bias Assessment

Data extraction and quality assessment were independently performed by two researchers and disagreements were resolved by consensus. We also tried to contact the original authors for missing information about the studies by emails, telephone, or fax whenever possible. The data consisted of the following items: 1) Basic information of the eligibility: The first author, nationality, publication year, and study design. 2) Basic characteristics of patients: Sample size, sex composition, average age, course of treatment, and the causes of HF of participants. 3) Details of interventions; 4) Details of outcomes. 5) Information of quality assessment of RCTs.

The methodological quality of included RCTs was assessed using the Cochrane risk-of-bias tool. The missing data was obtained from contacting the corresponding author via telephone, email, or fax. The quality assessment items of Cochrane tools included the following: 1) Selection bias: Random sequence generation and allocation concealment; 2) Performance bias: Blinding of the participants and personnel; 3) Detection bias: Blinding of the outcome assessment; 4) Attrition bias: Incomplete outcome data; 5) Reporting bias: Selective reporting; 6) Other bias. Each aspect was categorized into three levels: high risk, unclear risk, and low risk. Any disagreements were resolved by a third researcher.

### Data Analysis

Statistical analysis was performed using Cochrane Review Manager 5.3 (Copenhagen, The Nordic Cochrane Center, The Cochrane Collaboration, 2014). For dichotomous outcomes, the combined results were calculated as risk ratios (RRs) with 95% confidence intervals (95% CIs). For continuous outcomes, the Change Score was used to conduct the meta-analysis, and the Change Score was estimated according to the standard deviation (SD) of the pre-intervention data and post-intervention data by using the following formula provided by the Cochrane Handbook (16.1.3.2 Imputing standard deviations for changes from baseline):

$$SD_{change}=\sqrt{SD_{\text{pre}}^{2}+SD_{\text{post}}^{2}-\left(2\times Corr\times SD_{pre}\times SD\right)}\;Corr=0.8$$

The Change Scores were expressed as mean differences (MD) or standard mean differences (SMD) with 95% CIs. In this meta-analysis, the Change Scores of VPCs, LVEF, NT-proBNP, and QTd were presented as MD or SMD, while the total effective rate and ADRs were presented as RR. Heterogeneity across studies were assessed using the Cochrane I^2^ statistic. Data with low heterogeneity (I^2^ < 50%) was assessed as a fixed-effects model whereas others were assessed as a random-effects model. When data with high heterogeneity (I^2^>50%), sensitivity analysis was used to find the source of heterogeneity. The potential publication bias of outcomes was assessed by using Begg’s test. If the *p*-value of Begg’s test was lower than 0.05, there was publication bias among the studies.

## Results

### Study Selection

**FIGURE 1:**
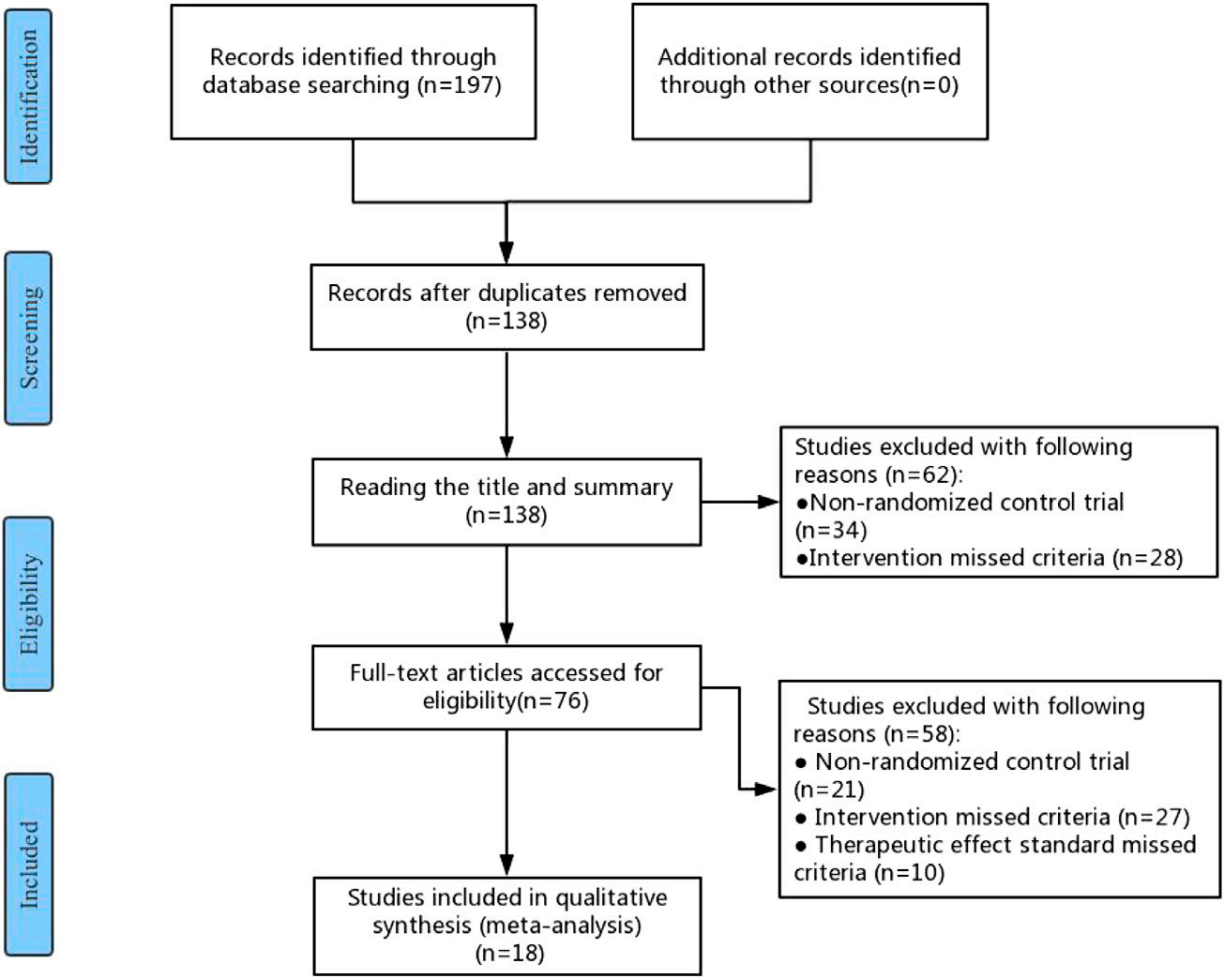
Flow chart of the search for eligible RCTs.

A total of 197 studies were identified in the initial literature search. After removing duplicates, 138 studies were retained for further examination. After screening the titles and abstracts, 62 studies were excluded because they were not RCTs (n = 34) or their interventions missed the criteria (n = 28). Further, 76 studies were eligible and examined, of which 58 were excluded due to the following reasons: 1) not RCTs (n = 21); 2) The interventions of RCTs did not meet the eligibility criteria (n = 27); 3) The effect standard did not meet the eligibility criteria (n = 10). Finally, 18 RCTs were eligible for this meta-analysis, which were carried out in China between 2010 and 2020 (He, 2010; Chen et al., 2011; Yang et al., 2012; Zhang et al., 2012; Song et al., 2014; Wan, 2014; Fang, 2016; Xue, 2016; Fang, 2017; Xu et al., 2017; Zeng et al., 2017; Hou, 2018; Wang, 2018; Zhao, 2018; He, 2019; Jing and Liu, 2019; Zhang, 2019; Li and Tan, 2020). The flow chart of the search strategy is shown in Figure 1.

### Study Characteristics

Eighteen RCTs with 1697 HF-VA patients accorded with the eligibility criteria, including 851 patients in the experimental groups and 846 patients in the control groups. 59.5% of the participants were male, and the majority was middle-aged and elderly. The causes of HF included coronary heart disease, hypertensive heart disease, dilated cardiomyopathy and valvular heart disease, of which coronary heart disease was the most common cause, accounting for 50.64%, followed by hypertensive heart disease (33.37%). There were no significant differences on general information between two groups. The patients in 55.6% of RCTs treated with amiodarone 0.2 g/time 3 times/day for 7–14 days, 0.2 g/time 2 times/day for next 7–14 days, and 0.1–0.2 g/time 1 time/day for final 7 days. The patients in 77.8% of RCTs treated with SSYX 1.6 g/time three times a day for the whole treatment course. The treatment course was 28 days in 77.8% of RCTs. The details of the study characteristics are depicted in Table 2. The detailed information of Chinese patent medicines of all the included studies are described in Supplementary File 3.

**TABLE 2:** Characteristics of the included studies.

Included studies	Sample size (E/C)	Sex (M/F)	Age(Y) (E/C)	CHD,HPD,DCM, VHD (E/C)	Intervention(E)	Intervention(C)	Course (days)	Outcomes
Wan (2014)	20/20	25/15	71.3 ± 4.6/72.1 ± 4.5	21,14,5,0	(Amiodarone 0.2 g Tid) + SSYX 1.6 g Tid	Amiodarone 0.2 g Tid	28	①
Fang (2016)	51/50	62/39	58.7 ± 6.4	61,30, 3,7	(Amiodarone 0.2 g Tid) plus SSYX 1.6 g Tid	(Amiodarone 0.2 g Tid)	56	①⑤⑥
Zhang et al. (2012)	32/32	45/19	63.0 ± 5.0/62.0 ± 8.0	23,17,3,5/22,15,5,6	(Amiodaron 0.2 g Tid 7days, 0.2 g Bid 7 days, 0.2 g Qd 14 days) + SSYX 1.6 g Tid	Amiodaron 0.2 g Tid 7 days, 0.2 g Bid 7 days, 0.2 g Qd 14 days	28	①②④
Li and Tan (2020)	50/50	69/31	57.7 ± 6.1/57.2 ± 6.6	NR	(Amiodaron 0.2 g Tid 7 days, 0.2 g Bid 7 days, 0.2 g Qd 14 days) plus SSYX 1.6 g Tid	Amiodaron 0.2 g Tid 7 days, 0.2 g Bid 7 days, 0.2 g Qd 14 days	28	①②③⑤⑥
Zhao (2018)	31/32	36/27	64.3 ± 2.9/63.2 ± 3.0	18,10,0,3/19,11,0,2	(Amiodaron 0.2 g Tid 7 days, 0.1 g Qd 21 days) plus SSYX 1.6 g Tid	Amiodaron 0.2 g Tid 7 days, 0.1 g Qd 21 days	28	①②
Chen et al. (2011)	60/56	NR	NR	NR	(Amiodaron 0.2 g Tid 7 days, 0.1 g Qd 21 days) plus SSYX 1.6 g Tid	Amiodaron 0.2 g Tid 7 days, 0.1 g Qd 21 days	28	①②⑥
Hou (2018)	75/75	91/59	56.5 ± 6.7/56.1 ± 6.2	NR	(Amiodaron 0.2 g Tid 7 days, 0.2 g Bid 7 days, 0.2 g Qd 14 days) plus SSYX 1.6 g Tid	Amiodaron 0.2 g Tid 7 days, 0.2 g Bid 7 days, 0.2 g Qd 14 days	28	①②③⑤⑥
Yang et al. (2012)	22/23	26/19	65.4/66.2	11,6,2,3/13,6,2,2	(Amiodaron 0.2 g Tid 7 days, 0.2 g Bid 7 days, 0.2 g Qd 14 days) plus SSYX 0.8 g Tid	Amiodaron 0.2 g Tid 7 days, 0.2 g Bid 7 days, 0.2 g Qd 14 days	28	①②
Xu et al. (2017)	47/47	53/41	64.7 ± 4.7	NR	(Amiodaron 0.2 g Tid 7 days, 0.2 g Bid 7 days, 0.2 g Qd 14 days) plus SSYX 1.6 g Tid	Amiodaron 0.2 g Tid 7 days, 0.2 g Bid 7 days, 0.2 g Qd 14 days	56	①②
Wang (2018)	50/50	55/45	51.3 ± 8.6/52.5 ± 9.3	NR	(Amiodaron 0.2 g Bid) plus SSYX 1.6 g Tid	Amiodaron 0.2 g Bid	28	①⑤⑥
Song et al. (2014)	66/66	81/51	59.2 ± 6.3	80,39,9,4	(Amiodaron 0.2 g Tid 7 days, 0.2 g Bid 7 days, 0.2 g Qd 14 days) plus SSYX 1.6 g Tid	Amiodaron 0.2 g Tid 7 days, 0.2 g Bid 7 days, 0.2 g Qd 14 days	56	①
Zhang (2019)	64/64	72/56	56.9 ± 4.1/57.1 ± 4.6	23,12,20,9/24,13,18,9	(Amiodaron 0.2 g Tid 7 days, 0.2 g Bid 7 days, 0.1 g Qd 70 days) plus SSYX 1.2 g Tid	Amiodaron 0.2 g Tid 7 days, 0.2 g Bid 7 days, 0.1 g Qd 70 days	84	①
He (2019)	43/43	48/38	66.9 ± 7.4/65.0 ± 5.2	NR	(Amiodaron 0.2 g Tid 7 days, 0.2 g Bid 7 days, 0.1 g Qd 14 days) plus SSYX 1.6 g Tid	Amiodaron 0.2 g Tid 7 days, 0.2 g Bid 7 days, 0.1 g Qd 14 days	28	②③⑥
Jin and Liu (2019)	35/35	40/30	57.4 ± 6.2/57.2 ± 6.6	14,21,0,0/13,12,10,0	(Amiodaron 0.2 g Tid 7 days, 0.2 g Bid 7 days, 0.2 g Qd 14 days) plus SSYX 1.6 g Tid	Amiodaron 0.2 g Tid 7 days, 0.2 g Bid 7 days, 0.2 g Qd 14 days	28	①②④
Zeng et al. (2017)	48/48	55/41	64.8 ± 8.0/65.2 ± 8.7	24,18,2,4/22,20,1,5	(Amiodaron 0.2 g Qd) plus SSYX 1.2 g Tid	Amiodaron 0.2 g Qd	84	①③⑤⑥
He (2010)	27/26	35/18	NR	NR	(Amiodaron 0.2 g Tid 7 days, 0.2 g Bid 7 days, 0.1 g Qd 14 days) plus SSYX 1.2 g Tid	Amiodaron 0.2 g Tid 7 days, 0.2 g Bid 7 days, 0.1 g Qd 14 days	28	①②
Xue (2016)	48/47	58/37	49.5 ± 4.2/50.3 ± 4.2	46,42,0,7	(Amiodaron 0.2 g Bid) plus SSYX 1.6 g Tid	Amiodaron 0.2 g Bid	28	②⑥
Fang (2017)	82/82	85/79	50.5 ± 4.3/51.5 ± 4.5	NR	(Amiodaron 0.2 g Bid) plus SSYX 1.6 g Tid	Amiodaron 0.2 g Bid	28	①⑤⑥

Note: E, experimental group; C, control group; M, male; F, female; Y, years; NR, not reported; CHD, coronary heart disease; HPD, hypertensive heart disease; DCM, dilated cardiomyopathy; VHD, valvular heart disease; SSYX, Shensong Yangxin capsule; Qd, one time a day; Bid, two times a day; Tid, three times a day; ①, effective rate; ②, adverse events; ③, the frequency of ventricular premature complexes; ④, left ventricular ejection fraction; ⑤, N terminal pro Btype natriuretic peptide; ⑥, QT dispersion.

### Quality Evaluation

**FIGURE 2:**
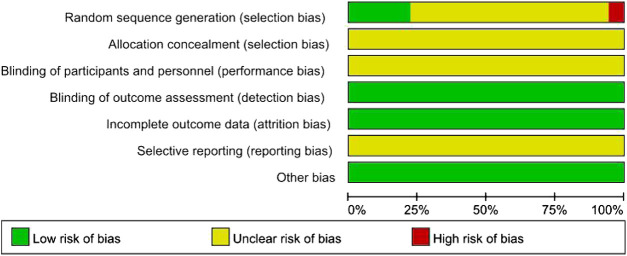
Risk-of-bias graph.

The Cochrane risk-of-bias assessment tool was used to evaluate the quality of each study. Further information of these studies was tried to obtain through contacting the correspondence authors via mail, telephone, or fax, however, none of them replied to our questions. 1) Selection bias (random sequence generation and allocation concealment): In one RCTs (Zeng et al., 2017), the patients were grouped according to the time of admission. Therefore, the risk of selection bias was considered “high”. In four RCTs (Xu et al., 2017; Hou, 2018; Wang, 2018; He, 2019), randomization was generated via random number table, therefore, the risk of selection bias was considered low. The remaining RCTs referred to only random grouping, and the risk of selection bias was considered “unclear”. Moreover, the information on allocation concealment was not observed in all included RCTs, so risk of selection bias was considered “unclear”. 2) Performance bias: All studies did not provide information on blinding, so the performance bias was evaluated as “unclear risk.” 3) Detection bias: The risk of detection bias was considered “low”, because the measurements of related results of the included RCTs were not affected by the blinding toward the outcome assessors. 4) Attrition bias: None of the included RCTs had incomplete data, so the risk of attrition bias was considered “low”. 5) Reporting bias: Considering that the complete implementation scheme could not be acquired, the risk of reporting bias was considered “unclear”. 6) Other bias: The risk of this bias was considered “low”, because no other obvious bias was observed in all studies. The quality assessment of the included RCTs is shown in Figure 2.

### Outcomes

#### The Total Effective Rate

**FIGURE 3:**
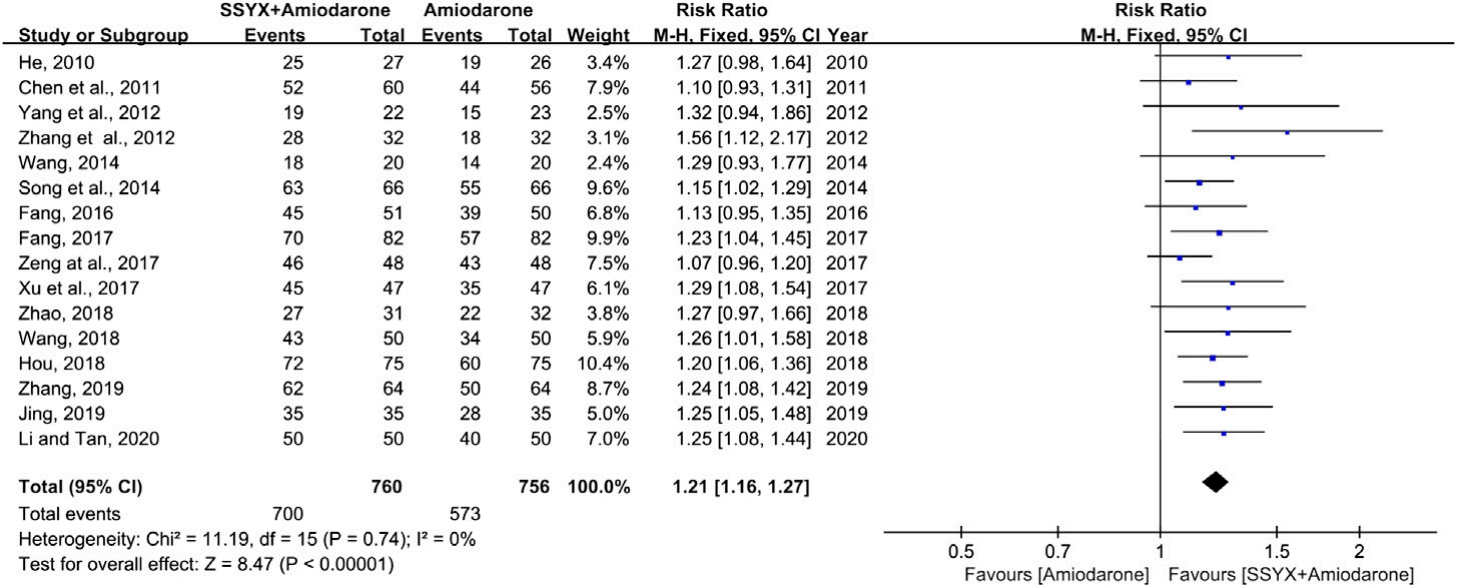
Forest plot of comparison: SSYX-amiodarone vs. amiodarone alone: The total effective rate.

Sixteen studies involving 1,516 participants adopted the effective rate to assess the clinical improvement (He, 2010; Chen et al., 2011; Yang et al., 2012; Zhang et al., 2012; Song et al., 2014; Wan, 2014; Fang, 2016; Fang, 2017; Xu et al., 2017; Zeng et al., 2017; Hou, 2018; Wang, 2018; Zhao, 2018; Jing and Liu, 2019; Zhang, 2019; Li and Tan, 2020). No statistically significant heterogeneity was found in analyses (I^2^ = 0%), and a fixed-effect model was used for statistical analysis. The meta-analysis results showed that SSYX-amiodarone could improve the total effective rate in patients with HF-VA compared with amiodarone alone (RR = 1.21; 95% CI (1.16, 1.27); *p* < 0.01), (Figure 3).

### ADRs

**FIGURE 4:**
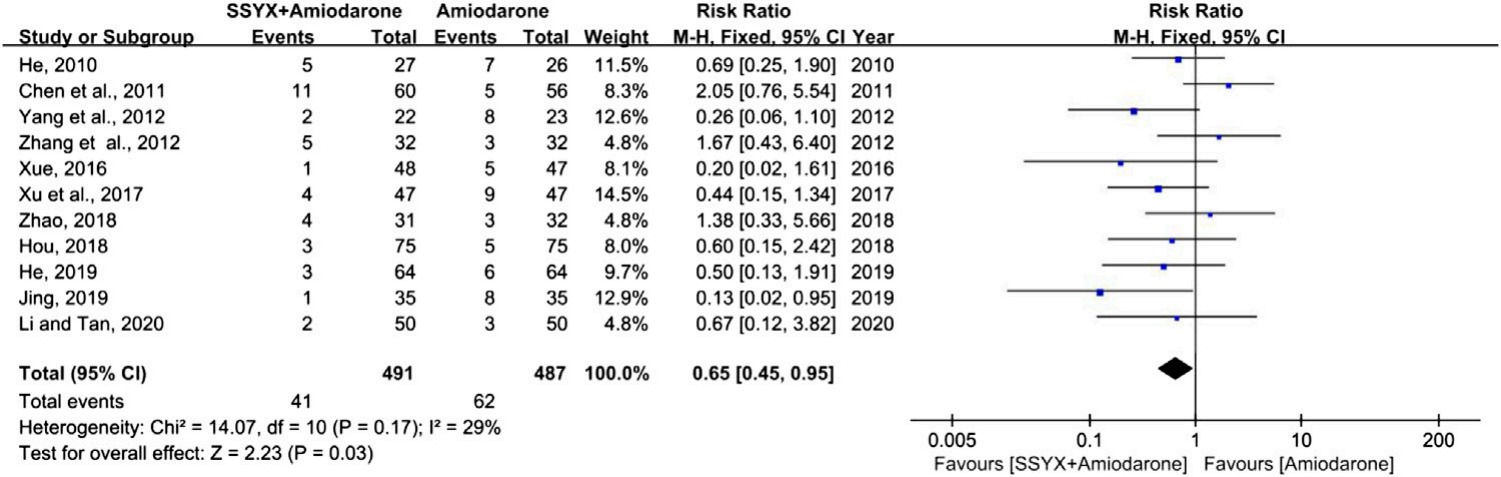
Forest plot of comparison: SSYX-amiodarone vs. amiodarone alone: ADRs.

Eleven studies involving 978 patients reported the ADRs (Chen et al., 2011; Hou, 2018; He, 2019; He, 2010; Jing and Liu, 2019; Li and Tan, 2020; Xu et al., 2017; Xue, 2016; Yang et al., 2012; Zhang et al., 2012; Zhao, 2018). Chest distress, nausea, dizziness, and sinus bradycardia were the common ADRs, while QT interval prolongation and liver damage were the rare ADRs. Low heterogeneity was found in analyses (I^2^ = 29%), thus a fixed-effect model was used for statistical analysis. The results showed that SSYX-amiodarone was safer than amiodarone alone in the treatment of HF-VA (RR = 0.65; 95% CI (0.45, 0.95); *p* = 0.03), (Figure 4).

### VPCs

**FIGURE 5:**
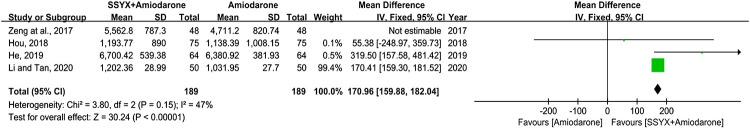
Forest plot of comparison: SSYX-amiodarone vs. amiodarone alone: VPCs.

Four studies involving 474 participants reported the VPCs (Li and Tan, 2020; Hou, 2018; He, 2019; Zeng et al., 2017). There was significant heterogeneity in the four studies (I^2^ = 86%), and a sensitivity analysis was conducted by excluding the studies one by one. The heterogeneity between the studies was significantly reduced after removing the study reported by Zeng et al. (I^2^ = 47%) (Zeng et al., 2017). As shown in Table 2, the amiodarone dose in the study “Zeng et al., 2017” was different from the other three studies, which might contribute to the heterogeneity. A fix-effect model was used for meta-analysis after removing the study “Zeng et al., 2017”. The results showed that SSYX-amiodarone was more effective in reducing the VPCs compared with amiodarone alone (MD = 170.96; 95% CI (159.88, 182.04); *p* < 0.01), (Figure 5).

### LVEF

**FIGURE 6:**
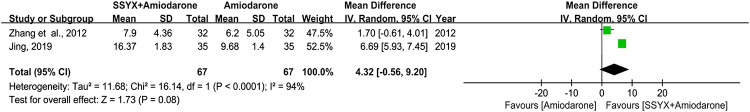
Forest plot of comparison: SSYX-amiodarone vs. amiodarone alone: LVEF.

Two studies involving 134 participants reported the LVEF (Zhang et al., 2012; Jing and Liu, 2019). Due to the high heterogeneity (I^2^ = 94%), the random-effect model was used for statistical analysis. The meta-analysis showed no significant difference between SSYX-amiodarone and amiodarone groups in enhancing LVEF (MD = 4.32; 95% CI (−0.56, 9.20); *p* = 0.08), (Figure 6).

### NT-proBNP

**FIGURE 7:**
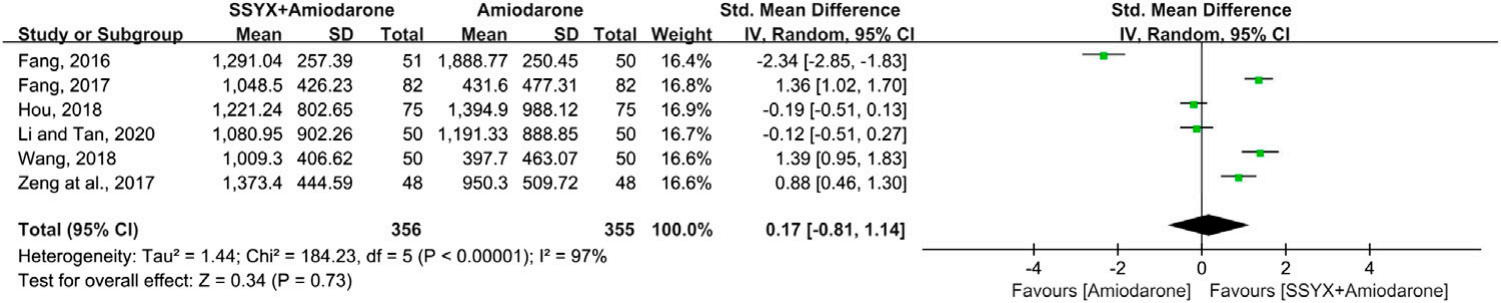
Forest plot of comparison: SSYX-amiodarone vs. amiodarone alone: NT-proBNP.

Six studies involving 711 patients reported the NT-proBNP (Fang, 2016; Fang, 2017; Zeng et al., 2017; Hou, 2018; Wang, 2018; Li and Tan, 2020). Significant heterogeneity was founded among the four studies (I^2^ = 97%) and the sensitivity analysis was performed by excluding the studies one by one. However, the heterogeneity could not be eliminated, thus a random-model was used for statistical analysis. The cause of high heterogeneity might be related tothe difference of usage and dosage of amiodarone among the six studies (Table 2). The results showed no significant difference between SSYX-amiodarone and amiodarone group in reducing the NT-proBNP (SMD = 0.17; 95% CI (-0.81, 1.14); *p* = 0.73), (Figure 7).

### QTd

**FIGURE 8:**
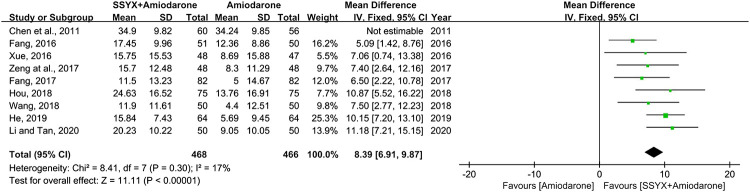
Forest plot of comparison: SSYX-amiodarone vs. amiodarone alone: QTd.

Nine studies involving 934 participants reported the QTd (Fang, 2016; Li and Tan, 2020; Chen et al., 2011; Hou, 2018; Wang, 2018; He, 2019; Zeng et al., 2017; Xue, 2016; Fang, 2017). There was significant heterogeneity among the nine studies (I^2^ = 66%), and the sensitivity analysis was carried out by excluding the studies one by one. The heterogeneity between the studies was significantly reduced after removing the study reported Chen et al. (I^2^ = 17%) (Chen et al., 2011). As shown in Table 2, the study “Chen et al., 2011” have not reported the gender and age of participants, while all the other studies have mentioned, and indicated there was not significant difference in above information between groups. Therefore, the cause of the heterogeneity might be related to the difference of gender and age of participants in the study “Chen et al., 2011”. A fix-effect model was used for meta-analysis after removing the study “Chen et al., 2011”. The results showed that SSYX-amiodarone was more effective in reducing the QTd compared with amiodarone alone (MD = 8.39; 95% CI (6.91, 9.87); *p* < 0.01), (Figure 8).

### Publication Bias of Outcomes

**FIGURE 9:**
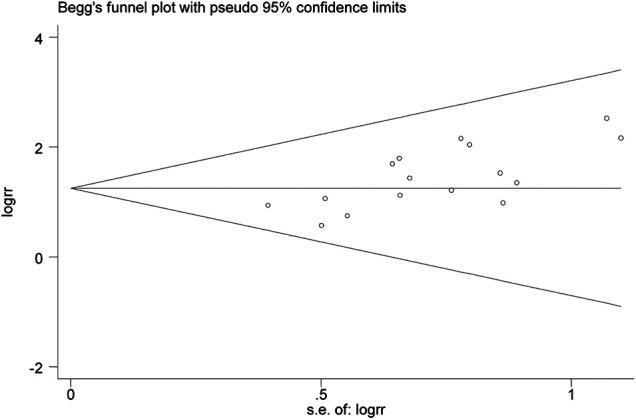
Begg’s funnel plot of the total effective rate. SSYX-amiodarone vs. amiodarone.

The publication bias was evaluated by Begg’s test. The *p*-value of Begg’s test of the total effective rate was 0.017 (*p* < 0.05), indicating publication bias among the studies. Besides, as shown in Figure 9, the Begg’s funnel plot of the total effective rate was not visually symmetrical, also indicating the existence of publication bias. The reason of the publication bias among studies might be that the sample size of the included RCTs was too small, and the lack of negative results might also be contributed to the bias.

## Discussion

### Summary of Evidences

This meta-analysis included 18 studies involving 1,697 participants. The results showed that SSYX-amiodarone had a better performance in improving the total effective rate, and reducing the ADRs, VPCs and QTd compared with amiodarone alone. Nevertheless, no significant differences of enhancing LVEF and reducing NT-proBNP were observed between SSYX-amiodarone and amiodarone groups. The main findings suggested that SSYX-amiodarone was better in improving VA compared with amiodarone alone, while there was no superiority in the improvement of HF.

The safety of traditional Chinese medicine has always been concerned. A total of 11 studies in this meta-analysis reported safety, and no serious ADRs were observed. The common side effects were chest tightness, nausea, dizziness and sinus bradycardia. QT interval prolongation and liver damage were occasionally reported. These discomforts could be effectively relieved after symptomatic treatment. The results of meta-analysis also showed that the safety of SSYX-amiodarone was better than those amiodarone alone. However, the end-point events including all-cause death and cardiovascular events in both SSYX-amiodarone and amiodarone alone groups were not observed and reported in all the included studies, which made it impossible to evaluate the long-term efficacy and safety of SSYX-amiodarone in the treatment of HF-VA.

Patient's conditions with HF-VA were often serious, while western medicine including Drug therapies and Non-drug therapies had limited efficacy. Amiodarone was a western medicine recommended by several guidelines for the treatment of HF-VA (Pedersen et al., 2014; Cao et al., 2020a). The innovative combination between amiodarone and traditional Chinese medicine in the treatment of HF-VA was a positive attempt, and some studies have shown that this combination had some advantages. For example, some studies (Chai et al., 2009a; Yang et al., 2013) showed that SSYX-amiodarone could normalize heart rate variability and heart rate turbulence, and reduced the incidence of ventricular tachycardia, suggesting that SSYX might provide electrophysiological benefits to patients with HF. SSYX was a new anti-arrhythmia drug developed under the guidance of the theory of venation, with “improving qi and nourishing Yin, activating blood and collaterals, clearing the heart and calming the spirit” as the treatment methods and “temperature, clarity, communication and tonic” as the prescription principles (Ma et al., 2016). Basic studies (Li et al., 2007a; Shi et al., 2009) have confirmed that SSYX could affect cardiac action potentials by blocking many ion channels, such as L-type calcium channel current, Na channel current, inward rectified potassium current and delayed rectified current, thus playing a broad-spectrum anti-arrhythmia effect. SSYX could inhibit Na channel current and L-type calcium channel current at different membrane potential levels, showing the effect of class I and IV antiarrhythmic drugs (Li et al., 2007b); At the same time, SSYX could reduce calcium overload in myocardial cells and played a protective role in myocardium (Gao and Li, 2014); SSYX could slow down the reactivation of the inactivated Kv1.4ΔN channel by suppressing the peak current (Wang et al., 2009); SSYX had a significant inhibitory effect on hHCN4 current, thus playing a role in the treatment of ventricular premature beats (Sun et al., 2010). At the same time, SSYX also had a non-ion channel regulation effect. For example, SSYX could improve the myocardial conduction function by promoting the pulse conduction in the sinoatrial node, atrium and atrioventricular node (Jin et al., 2009); Meanwhile, SSYX could improve the electrophysiological matrix of the ventricle by inhibiting ventricular remodeling, and reduce the dispersion of action potential duration in different parts of the ventricle, which was beneficial to eliminate reentrant and reduce the occurrence of arrhythmia (Chai et al., 2009b; Feng et al., 2009; Shen et al., 2014); Additionally, researchers also found the regulatory effect of SSYX on the cardiac autonomic nerve function by inhibiting the neural remodeling after myocardial infarction, thus playing an anti-arrhythmia role (Jiang et al., 2014).

Recently, a meta-analysis similar to our study was evaluated the efficacy and safety of SSYX-amiodarone in the treatment of HF with arrhythmia (Tian, et al., 2020). Tian's research included patients with HF complicated by atrial premature beat, atrial fibrillation, sinus arrhythmia and ventricular premature beat, while our research focused on patients with HF-VA. The deadline of Tian's research search was October 2018, while the deadline of our research was June 2020. Four studies included in our meta-analysis were published from October 2018 to June 2020. In addition, there were some other differences in methodology and selection of outcome indicators between our study and Tian's study. In conclusion, our study was different from the previous published meta-analysis in the topic and content of research. Through our study, the efficacy and safety of SSYX-amiodarone in the treatment of HF-VA could be comprehensively evaluated.

### Limitations

Several potential limitations should be mentioned in this meta-analysis. First, the quality of the included RCTs was poor. Despite all patients reported in the trials were randomly assigned into different groups, only four RCTs described the methods of generating random sequences, such as a random number table. No studies mentioned blinding for researchers or participants. These contributed to an exaggerated curative effect and decreased reliability of the evidence. Second, the risk of publication bias was so high that it might affect the strength of the results. Most of the trials were small sample studies with positive findings. All included trials were published in Chinese. Finally, the International Committee of Medical Journal Editors published a statement in September 2004 requiring that all clinical trials must be registered to be considered for publication (DeAngelis et al., 2006). However, all included studies of this meta-analysis have not been registered.

### Implication

The significance of this meta-analysis might be more to identify current problems and areas worthy of improvement. Due to the poor methodological quality of the included studies, the evidence provided by this meta-analysis was still insufficient to support the routine use of SSYX-amiodarone for HF-VA. The validity of the conclusion of meta-analysis was highly dependent on the quality of the RCTs included, thus the importance of the quality assessment of individual RCT must be emphasized again. There were some recommendations for further studies on SSYX-amiodarone in the treatment of HF-VA: 1) Clinical trials should be prospectively registered in international clinical trials registry platform. 2) The quality of study designs including randomization, allocation concealment, and blinding should be improved. 3) Consistency in outcome measures should be paid more attention. 4) The end-point events of SSYX-amiodarone for the treatment of HF-VA should be observed.

## Conclusion

The findings of this meta-analysis suggested SSYX-amiodarone seemed to be more effective and safer than amiodarone alone in the treatment of HF-VA. However, because of the high risk of bias and low quality of the included trials, we might be unable to draw any conclusions about its routine use. The overall effect still needs to be verified through more well-design clinical studies with reasonable sample and good methodological quality in further.

## Data Availability

The original contributions presented in the study are included in the article/Supplementary Material, further inquiries can be directed to the corresponding author.
