# Supplementary material for: Clinical Efficacy and Safety of Shensong Yangxin Capsule-Amiodarone Combination on Heart Failure Complicated by Ventricular Arrhythmia: A Meta-Analysis of Randomized Controlled Trials
Source: Front Pharmacol. 2021 Feb 22;12:613922. doi: 10.3389/fphar.2021.613922 (PMC7937972; doi:10.3389/fphar.2021.613922)
Supplement: Supplementary file 2 [file datasheet2.docx]

***Supplementary file 2*** The details of search terms and strategies

Take searching PubMed as an example, the search terms and strategies are as follows:

#1 Heart failure [MeSH Terms]

#2 Heart failure [Title/Abstract]

#3 Ventricular arrhythmia [MeSH Terms]

#4 Ventricular arrhythmia [Title/Abstract]

#5 premature ventricular beat [MeSH Terms]

#6 premature ventricular beat [Title/Abstract]

#7 ventricular tachycardia [MeSH Terms]

#8 ventricular tachycardia [Title/Abstract]

#9 ventricular flutter [MeSH Terms]

#10 ventricular flutter [Title/Abstract]

#11 ventricular fibrillation [MeSH Terms]

#12 ventricular fibrillation [Title/Abstract]

#13 #1 OR #2 OR #3 OR #4 #5 OR #6 OR #7 OR #8 #9 OR #10 OR #11 OR #12

#14 ShensongYangxin capsule[MeSH Terms]

#15 Shensong Yangxin capsule [MeSH Terms]

#16 Shensong Yangxin [MeSH Terms]

#17 ShensongYangxin capsule [Title/Abstract]

#18 Shensong Yangxin capsule [Title/Abstract]

#19 Shensong Yangxin [Title/Abstract]

#20 Chinese patent medicine [Title/Abstract]

#21 Chinese herb medicine [Title/Abstract]

#22 Traditional Chinese medicine [Title/Abstract]

#23 #14 OR #15 OR#16 OR #17 OR #18 OR #19 OR#20 OR #21 OR #22

#24 Randomized Controlled Trial [Publication Type]

#25 Controlled Clinical Trial [Publication Type]

#26 random* [All Fields]

#27 #24 OR #25 OR #26

#28 #13AND #23 AND #27 ”
