# Supplementary material for: Clinical Efficacy and Safety of Shensong Yangxin Capsule-Amiodarone Combination on Heart Failure Complicated by Ventricular Arrhythmia: A Meta-Analysis of Randomized Controlled Trials
Source: Front Pharmacol. 2021 Feb 22;12:613922. doi: 10.3389/fphar.2021.613922 (PMC7937972; doi:10.3389/fphar.2021.613922)
Supplement: Supplementary file 3 [file datasheet3.docx]

***Supplementary file 3***  The details of Chinese patent medicines of all the included studies

| **Study** | **Formu**  **lation** | **Source** | **Species** | **Quality control reported?  (Y/N)** | **Chemical analysis reported?(Y/N)** |
| --- | --- | --- | --- | --- | --- |
| Wang, 2014 | ShenSong YangXin Capsule | Beijing YiLing Pharmaceutical Co., Ltd | Root of *Panax ginseng* C.A.Mey., Root of *Ophiopogon japonicus* (Thunb.) Ker Gawl., Flesh of *Cornus oj-jZcinalis Sieb.* etZucc., .Root of *Taxillus chinensis* (DC.) Danser., Whole worm of *Eupolyphaga sinensis Walke.,* Root of *Paeonia veitchii* Lynch., Root of *Coptis chinensis* Franch., Coptis deltoidea., Fruit of *Schisandra sphenanthera* Rehder & E.H.Wils., Fossils of bones of *elephant, rhinoceros or horse* | Y-Prepared according to Chinese pharmacopeia(2010) | Y-HPLC |
| Fang, 2016 | ShenSong YangXin Capsule | Beijing YiLing Pharmaceutical Co., Ltd | Root of *Panax ginseng* C.A.Mey., Root of *Ophiopogon japonicus* (Thunb.) Ker Gawl., Flesh of *Cornus oj-jZcinalis Sieb.* etZucc., .Root of *Taxillus chinensis* (DC.) Danser., Whole worm of *Eupolyphaga sinensis Walke.,* Root of *Paeonia veitchii* Lynch., Root of *Coptis chinensis* Franch., Coptis deltoidea., Fruit of *Schisandra sphenanthera* Rehder & E.H.Wils., Fossils of bones of *elephant, rhinoceros or horse* | Y-Prepared according to Chinese pharmacopeia(2015) | Y-HPLC |
| Zhang et al., 2012 | ShenSong YangXin Capsule | Shijiazhuang YiLing Pharmaceutical Co., Ltd | Root of *Panax ginseng* C.A.Mey., Root of *Ophiopogon japonicus* (Thunb.) Ker Gawl., Flesh of *Cornus oj-jZcinalis Sieb.* etZucc., .Root of *Taxillus chinensis* (DC.) Danser., Whole worm of *Eupolyphaga sinensis Walke.,* Root of *Paeonia veitchii* Lynch., Root of *Coptis chinensis* Franch., Coptis deltoidea., Fruit of *Schisandra sphenanthera* Rehder & E.H.Wils., Fossils of bones of *elephant, rhinoceros or horse*. | Y-Prepared according to Chinese pharmacopeia(2010) | Y-HPLC |
| Li and Tan, 2020 | ShenSong YangXin Capsule | Not mentioned | Root of *Panax ginseng* C.A.Mey., Root of *Ophiopogon japonicus* (Thunb.) Ker Gawl., Flesh of *Cornus oj-jZcinalis Sieb.* etZucc., .Root of *Taxillus chinensis* (DC.) Danser., Whole worm of *Eupolyphaga sinensis Walke.,* Root of *Paeonia veitchii* Lynch., Root of *Coptis chinensis* Franch., Coptis deltoidea., Fruit of *Schisandra sphenanthera* Rehder & E.H.Wils., Fossils of bones of *elephant, rhinoceros or horse* | - | - |
| Zhao, 2018 | ShenSong YangXin Capsule | YiLing Pharmaceutical Co., Ltd | Root of *Panax ginseng* C.A.Mey., Root of *Ophiopogon japonicus* (Thunb.) Ker Gawl., Flesh of *Cornus oj-jZcinalis Sieb.* etZucc., .Root of *Taxillus chinensis* (DC.) Danser., Whole worm of *Eupolyphaga sinensis Walke.,* Root of *Paeonia veitchii* Lynch., Root of *Coptis chinensis* Franch., Coptis deltoidea., Fruit of *Schisandra sphenanthera* Rehder & E.H.Wils., Fossils of bones of *elephant, rhinoceros or horse* | Y-Prepared according to Chinese pharmacopeia(2015) | Y-HPLC |
| Chen et al., 2011 | ShenSong YangXin Capsule | Shijaizhuang YiLing Pharmaceutical Co., Ltd | Root of *Panax ginseng* C.A.Mey., Root of *Ophiopogon japonicus* (Thunb.) Ker Gawl., Flesh of *Cornus oj-jZcinalis Sieb.* etZucc., .Root of *Taxillus chinensis* (DC.) Danser., Whole worm of *Eupolyphaga sinensis Walke.,* Root of *Paeonia veitchii* Lynch., Root of *Coptis chinensis* Franch., Coptis deltoidea., Fruit of *Schisandra sphenanthera* Rehder & E.H.Wils., Fossils of bones of *elephant, rhinoceros or horse* | Y-Prepared according to Chinese pharmacopeia(2010) | Y-HPLC |
| Hou, 2018 | ShenSong YangXin Capsule | Beijing YiLing Pharmaceutical Co., Ltd | Root of *Panax ginseng* C.A.Mey., Root of *Ophiopogon japonicus* (Thunb.) Ker Gawl., Flesh of *Cornus oj-jZcinalis Sieb.* etZucc., .Root of *Taxillus chinensis* (DC.) Danser., Whole worm of *Eupolyphaga sinensis Walke.,* Root of *Paeonia veitchii* Lynch., Root of *Coptis chinensis* Franch., Coptis deltoidea., Fruit of *Schisandra sphenanthera* Rehder & E.H.Wils., Fossils of bones of *elephant, rhinoceros or horse* | Y-Prepared according to Chinese pharmacopeia(2015) | Y-HPLC |
| Yang et al., 2012 | ShenSong YangXin Capsule | Shijiazhuang YiLing Pharmaceutical Co., Ltd | Root of *Panax ginseng* C.A.Mey., Root of *Ophiopogon japonicus* (Thunb.) Ker Gawl., Flesh of *Cornus oj-jZcinalis Sieb.* etZucc., .Root of *Taxillus chinensis* (DC.) Danser., Whole worm of *Eupolyphaga sinensis Walke.,* Root of *Paeonia veitchii* Lynch., Root of *Coptis chinensis* Franch., Coptis deltoidea., Fruit of *Schisandra sphenanthera* Rehder & E.H.Wils., Fossils of bones of *elephant, rhinoceros or horse* | Y-Prepared according to Chinese pharmacopeia(2010) | Y-HPLC |
| Xu et al., 2017 | ShenSong YangXin Capsule | Not mentioned | Root of *Panax ginseng* C.A.Mey., Root of *Ophiopogon japonicus* (Thunb.) Ker Gawl., Flesh of *Cornus oj-jZcinalis Sieb.* etZucc., .Root of *Taxillus chinensis* (DC.) Danser., Whole worm of *Eupolyphaga sinensis Walke.,* Root of *Paeonia veitchii* Lynch., Root of *Coptis chinensis* Franch., Coptis deltoidea., Fruit of *Schisandra sphenanthera* Rehder & E.H.Wils., Fossils of bones of *elephant, rhinoceros or horse* | - | - |
| Wang, 2018 | ShenSong YangXin Capsule | Beijing YiLing Pharmaceutical Co., Ltd | Root of *Panax ginseng* C.A.Mey., Root of *Ophiopogon japonicus* (Thunb.) Ker Gawl., Flesh of *Cornus oj-jZcinalis Sieb.* etZucc., .Root of *Taxillus chinensis* (DC.) Danser., Whole worm of *Eupolyphaga sinensis Walke.,* Root of *Paeonia veitchii* Lynch., Root of *Coptis chinensis* Franch., Coptis deltoidea., Fruit of *Schisandra sphenanthera* Rehder & E.H.Wils., Fossils of bones of *elephant, rhinoceros or horse* | Y-Prepared according to Chinese pharmacopeia(2015) | Y-HPLC |
| Song et al., 2014 | ShenSong YangXin Capsule | Not mentioned | Root of *Panax ginseng* C.A.Mey., Root of *Ophiopogon japonicus* (Thunb.) Ker Gawl., Flesh of *Cornus oj-jZcinalis Sieb.* etZucc., .Root of *Taxillus chinensis* (DC.) Danser., Whole worm of *Eupolyphaga sinensis Walke.,* Root of *Paeonia veitchii* Lynch., Root of *Coptis chinensis* Franch., Coptis deltoidea., Fruit of *Schisandra sphenanthera* Rehder & E.H.Wils., Fossils of bones of *elephant, rhinoceros or horse* | - | - |
| Zhang, 2019 | ShenSong YangXin Capsule | Beijing YiLing Pharmaceutical Co., Ltd | Root of *Panax ginseng* C.A.Mey., Root of *Ophiopogon japonicus* (Thunb.) Ker Gawl., Flesh of *Cornus oj-jZcinalis Sieb.* etZucc., .Root of *Taxillus chinensis* (DC.) Danser., Whole worm of *Eupolyphaga sinensis Walke.,* Root of *Paeonia veitchii* Lynch., Root of *Coptis chinensis* Franch., Coptis deltoidea., Fruit of *Schisandra sphenanthera* Rehder & E.H.Wils., Fossils of bones of *elephant, rhinoceros or horse* | Y-Prepared according to Chinese pharmacopeia(2015) | Y-HPLC |
| He, 2019 | ShenSong YangXin Capsule | Yi Ling pharmaceutical factory | Root of *Panax ginseng* C.A.Mey., Root of *Ophiopogon japonicus* (Thunb.) Ker Gawl., Flesh of *Cornus oj-jZcinalis Sieb.* etZucc., .Root of *Taxillus chinensis* (DC.) Danser., Whole worm of *Eupolyphaga sinensis Walke.,* Root of *Paeonia veitchii* Lynch., Root of *Coptis chinensis* Franch., Coptis deltoidea., Fruit of *Schisandra sphenanthera* Rehder & E.H.Wils., Fossils of bones of *elephant, rhinoceros or horse* | Y-Prepared according to Chinese pharmacopeia(2015) | Y-HPLC |
| Jin and Liu,  2019 | ShenSong YangXin Capsule | Not mentioned | Root of *Panax ginseng* C.A.Mey., Root of *Ophiopogon japonicus* (Thunb.) Ker Gawl., Flesh of *Cornus oj-jZcinalis Sieb.* etZucc., .Root of *Taxillus chinensis* (DC.) Danser., Whole worm of *Eupolyphaga sinensis Walke.,* Root of *Paeonia veitchii* Lynch., Root of *Coptis chinensis* Franch., Coptis deltoidea., Fruit of *Schisandra sphenanthera* Rehder & E.H.Wils., Fossils of bones of *elephant, rhinoceros or horse* | - | - |
| Zeng et al., 2017 | ShenSong YangXin Capsule | Beijing YiLing Pharmaceutical Co., Ltd | Root of *Panax ginseng* C.A.Mey., Root of *Ophiopogon japonicus* (Thunb.) Ker Gawl., Flesh of *Cornus oj-jZcinalis Sieb.* etZucc., .Root of *Taxillus chinensis* (DC.) Danser., Whole worm of *Eupolyphaga sinensis Walke.,* Root of *Paeonia veitchii* Lynch., Root of *Coptis chinensis* Franch., Coptis deltoidea., Fruit of *Schisandra sphenanthera* Rehder & E.H.Wils., Fossils of bones of *elephant, rhinoceros or horse* | Y-Prepared according to Chinese pharmacopeia(2015) | Y-HPLC |
| He, 2010 | ShenSong YangXin Capsule | Not mentioned | Root of *Panax ginseng* C.A.Mey., Root of *Ophiopogon japonicus* (Thunb.) Ker Gawl., Flesh of *Cornus oj-jZcinalis Sieb.* etZucc., .Root of *Taxillus chinensis* (DC.) Danser., Whole worm of *Eupolyphaga sinensis Walke.,* Root of *Paeonia veitchii* Lynch., Root of *Coptis chinensis* Franch., Coptis deltoidea., Fruit of *Schisandra sphenanthera* Rehder & E.H.Wils., Fossils of bones of *elephant, rhinoceros or horse* | - | - |
| Xue, 2016 | ShenSong YangXin Capsule | Beijing YiLing Pharmaceutical Co., Ltd | Root of *Panax ginseng* C.A.Mey., Root of *Ophiopogon japonicus* (Thunb.) Ker Gawl., Flesh of *Cornus oj-jZcinalis Sieb.* etZucc., Root of *Taxillus chinensis* (DC.) Danser., Whole worm of *Eupolyphaga sinensis Walke.,* Root of *Paeonia veitchii* Lynch., Root of *Coptis chinensis* Franch., Coptis deltoidea., Fruit of *Schisandra sphenanthera* Rehder & E.H.Wils., Fossils of bones of *elephant, rhinoceros or horse* | Y-Prepared according to Chinese pharmacopeia(2015) | Y-HPLC |
| Fang, 2017 | ShenSong YangXin Capsule | Beijing YiLing Pharmaceutical Co., Ltd | Root of *Panax ginseng* C.A.Mey., Root of *Ophiopogon japonicus* (Thunb.) Ker Gawl., Flesh of *Cornus oj-jZcinalis Sieb.* etZucc., .Root of *Taxillus chinensis* (DC.) Danser., Whole worm of *Eupolyphaga sinensis Walke.,* Root of *Paeonia veitchii* Lynch., Root of *Coptis chinensis* Franch., Coptis deltoidea., Fruit of *Schisandra sphenanthera* Rehder & E.H.Wils., Fossils of bones of *elephant, rhinoceros or horse* | Y-Prepared according to Chinese pharmacopeia(2015) | Y-HPLC |
